# Investigating metamemory and its neural correlates in item‐ and associative memory in older adults with mild cognitive impairment

**DOI:** 10.1002/alz70856_102293

**Published:** 2025-12-25

**Authors:** Raphael Gabiazon, Jasmine Wright, Samantha Marshall, Gianna Jeyarajan, Jennifer Hanna Al‐Shaikh, Lindsay S Nagamatsu

**Affiliations:** ^1^ Schulich School of Medicine & Dentistry, London, ON, Canada; ^2^ Western University, London, ON, Canada

## Abstract

**Background:**

Older adults with mild cognitive impairment (MCI) often exhibit greater memory decline than expected for their age, increasing their susceptibility to Alzheimer's disease (AD). Among these deficits, associative memory—remembering pairs of unrelated items—appears particularly vulnerable and may reflect broader deficits in item‐based processing. Although such deficits are frequently attributed to biological factors, evidence suggests they may also be influenced by metamemory, which encompasses perceptions, beliefs, and judgments about one's own memory. Metamemory has been shown to influence memory, but its role at both the item and associative levels, and their corresponding neural correlates in MCI, remains unclear. Therefore, we conducted a cross‐sectional fMRI study to examine how different metamemory beliefs affect item‐ and associative‐level recognition in MCI.

**Method:**

Twenty‐eight MCI participants (60–80 years; mean=72.61; 53.57% female) completed three scales of the Multifactorial Memory Questionnaire (MMQ) (satisfaction, ability, and strategies). Participants underwent a mixed block/event‐related associative memory task in fMRI consisting of three runs with randomized encoding/recognition of faces, places, and face–place pairs. Behavioural measures were d′ and reaction time (RT) for each recognition condition.

**Result:**

Cluster‐corrected whole‐brain analysis (z>1.65, *p* <.05) identified involvement of the intracalcarine cortex, occipital pole, lingual gyrus, and lateral occipital cortex. Partial correlations controlling for age and sex showed higher d′ for place was related to faster RTs for faces (r=–0.61, *p* = .0005), places (r=–0.53, *p* = .0037), and face–place pairs (r=–0.54, *p* = .0028). Satisfaction correlated positively with self‐appraised ability (r=0.51, *p* = .006) but negatively with reported strategies (r=–0.40, *p* = .033) and occipital pole activation (r=–0.41, *p* = .048). Two‐way (Group × Condition) ANOVAs were conducted after median splits on each MMQ scale (High vs. Low), revealing main effects of Condition on d′ (F(2,52)=5.79, *p* = .005, η^2^=.18) and RT (F(2,52)=36.52, *p* <.001, η^2^=.58). Face recognition was most accurate (*p* = .008) and fastest (*p* <.001), with place intermediate. Only self‐reported strategies showed a main Group effect (F(1,26)=8.04, *p* = .009, η^2^=.24), with low‐strategy participants outperforming high‐strategy users.

**Conclusion:**

Our findings suggest that metamemory impacts recognition performance and neural activity in MCI, with associative memory especially vulnerable. Incorporating metamemory measures in clinical protocols could improve diagnostic precision and help manage AD risk.

